# Popliteal cystic adventitial disease unmasked by intravascular ultrasound

**DOI:** 10.1016/j.jvscit.2026.102279

**Published:** 2026-04-29

**Authors:** Natasha Hasemaki, Michael Lichtenberg, Konstantinos Stavroulakis

**Affiliations:** a Department of Vascular Surgery, University Hospital LMU Munich, Munich, Germany; b Angiology Department, Klinikum Hochsauerland, Arnsberg, Germany; c Vascular and Endovascular Surgery Unit, Mathias Spital Rheine, Rheine, Germany

**Keywords:** Cystic adventitial disease, Intravascular ultrasound, Popliteal artery

## Abstract

Cystic adventitial disease (CAD) of the popliteal artery is a rare, nonatherosclerotic cause of intermittent claudication that often mimics peripheral arterial disease, leading to misdiagnosis. We report a 60-year-old man with progressive claudication and duplex evidence of popliteal occlusion. After lesion crossing, intravascular ultrasound (IVUS) revealed a cystic intramural lesion consistent with CAD, prompting surgical repair with complete symptom resolution. A focused literature review identified five IVUS-diagnosed cases, predominantly in middle-aged men. Conventional imaging frequently failed to establish the diagnosis. IVUS reliably identified CAD and guided appropriate management when standard imaging was inconclusive.

Cystic adventitial disease (CAD) of the popliteal artery is a rare and often under-recognized cause of lower-limb intermittent claudication (IC), typically affecting middle-aged men without the usual atherosclerotic risk factors.^1^, ^2^, ^3^ It is estimated to account for approximately 0.1% of all vascular diseases and about one in 1200 cases of IC.^2^^,^^3^ Characterized by mucinous cyst formation within the arterial adventitia, CAD can mimic peripheral artery disease (PAD), leading to diagnostic challenges and potential mismanagement. Although the precise pathogenesis of CAD is unclear, several mechanisms have been suggested, including recurrent microtrauma, congenital or developmental defects, migration of ganglion cysts from neighboring joints, and the articular theory, which associates cyst formation with synovial structures of the knee.^4^

Clinically, CAD can closely resemble atherosclerotic PAD, manifesting as IC, reduced distal pulses, and low ankle–brachial index (ABI) values, which often leads to misdiagnosis and inappropriate management. Although conventional imaging modalities such as duplex ultrasonography and digital subtraction angiography (DSA) provide useful information, they may fail to fully characterize the lesion, especially in cases of total occlusion. Although magnetic resonance imaging (MRI) is regarded as the gold standard for defining cyst morphology,^5^ extent, and potential joint communication, MRI is not routinely used in PAD evaluation, and its availability may be limited in acute settings.

Recently, intravascular ultrasound (IVUS) has been increasingly incorporated into the armamentarium for PAD treatment. Several studies^6^, ^7^, ^8^ have demonstrated that its use can improve the outcomes of endovascular therapy and reduce the need for reinterventions and major adverse limb events.^9^ One of the main reasons is that IVUS provides a better understanding of the underlying disease, distinguishing between subintimal and intraluminal passage, facilitating accurate vessel sizing and stent selection, and enabling the detection of periprocedural complications. However, its diagnostic application in rare, nonatherosclerotic causes of claudication remains infrequently reported.

Herein, we present a case of popliteal artery CAD initially presumed to be an atherosclerotic occlusion, in which IVUS served as an intraprocedural adjunct that aided diagnostic clarification and guided appropriate surgical management. In addition, we performed a focused review of the current literature to synthesize evidence on the diagnostic utility of IVUS in CAD and its implications for treatment.

## Case report

**Fig 1:**
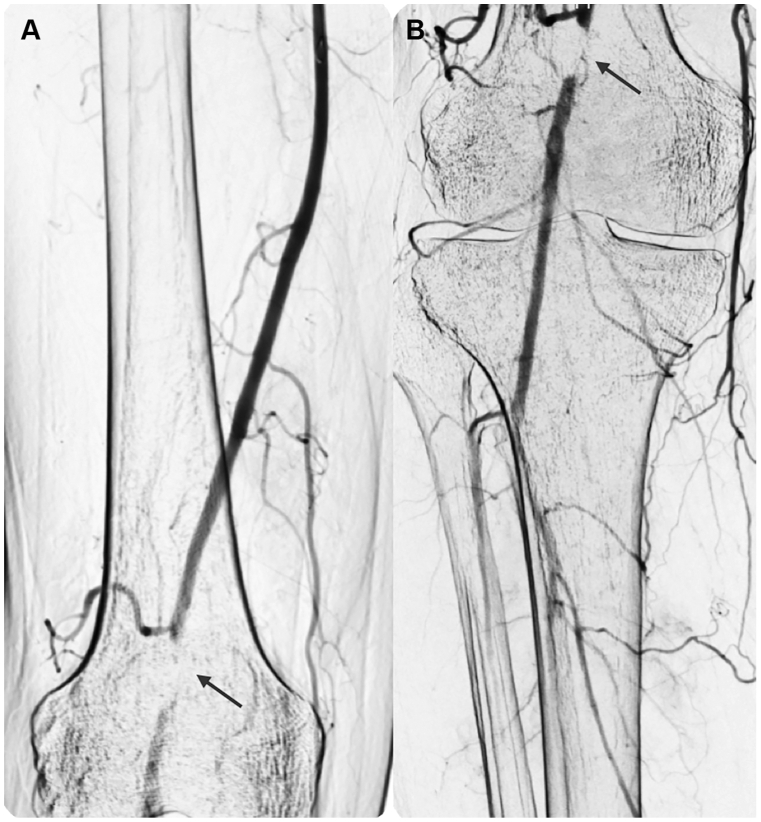
Intraoperative digital subtraction angiography (DSA) of femoropopliteal **(A)** and infrapopliteal segment **(B)** confirmed a short-segment chronic total occlusion of the popliteal artery at the P2 level (*black arrow*).

**Fig 2:**
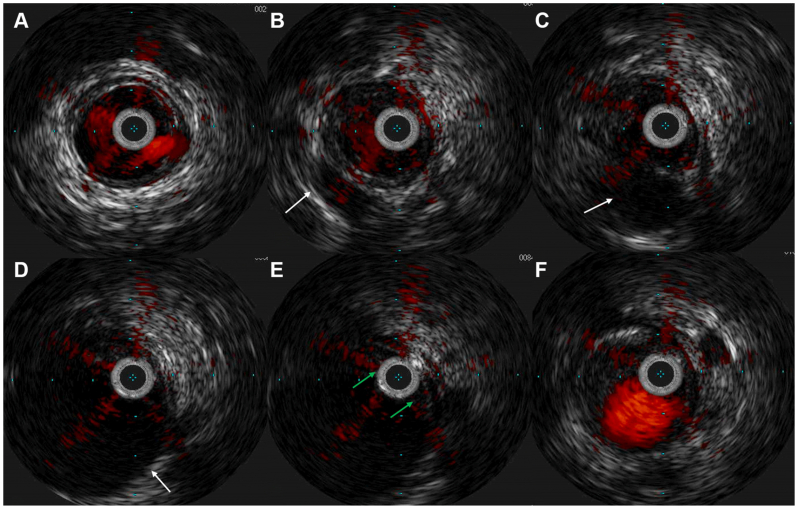
Intravascular ultrasound (IVUS) cross-sectional images of the popliteal artery from proximal to distal. **(A)** Proximal popliteal artery demonstrating the typical three-layered appearance of the arterial wall. **(B**-**D)** Progressive eccentric compression of the arterial lumen by a crescent-shaped cyst (*white arrows*). **(E)** Complete luminal compression caused by the cyst (*green arrows*). **(F)** Distal popliteal artery with preserved three-layered wall structure.

A 60-year-old man presented to the Angiology and Vascular Medicine Center of Arnsberg with a 6-month history of progressive IC of the right leg, limiting his walking distance to approximately 200 m (stage 3 according to Rutherford classification). He denied any history of trauma. Apart from a history of smoking, he had no significant cardiovascular risk factors. There were no signs of cyanosis, ulceration, or trophic changes in either limb. Physical examination revealed diminished right popliteal and distal pulses, with normal pulses in the left lower extremity, whereas the ABI was 0.8 on the right and 1.1 on the left lower limb. Routine laboratory tests, including biochemical profile and inflammatory markers, were within normal limits. Duplex ultrasonography (DUS) demonstrated occlusion of the right popliteal artery, appearing as hypoechoic intraluminal material suggestive of thrombus. DSA was performed to confirm the presence and extent of the popliteal artery occlusion, evaluate distal runoff, and assess the feasibility of endovascular treatment. Baseline angiography confirmed a short-segment chronic total occlusion of the popliteal artery at the P2 level. The characteristic “scimitar sign,” which has been described in CAD, was not evident on angiography in our case, which further contributed to the initial assumption of atherosclerotic occlusion. The proximal superficial femoral artery, as well as the distal tibioperoneal trunk and anterior tibial artery, showed no evidence of atherosclerosis (Fig 1). An attempt was made to cross the lesion using a 0.018-inch Advantage guidewire (Terumo Corp) in combination with a Navicross catheter (Terumo Corp). The guidewire crossed the lesion easily without significant resistance, suggesting passage through a compressed lumen rather than a heavily calcified occlusion. Given the atypical angiographic appearance and the absence of significant atherosclerotic disease in adjacent arterial segments, IVUS was performed prior to any endovascular treatment to better characterize the lesion morphology, using Visions PV .018 digital catheter (Philips Volcano). IVUS demonstrated an eccentric, hypoechoic lesion within the arterial wall, involving over one-half of the vessel circumference. The intimal layer was preserved without evidence of plaque, calcification, or thrombus. These features were consistent with a cystic adventitial lesion. (Fig 2).

**Fig 3:**
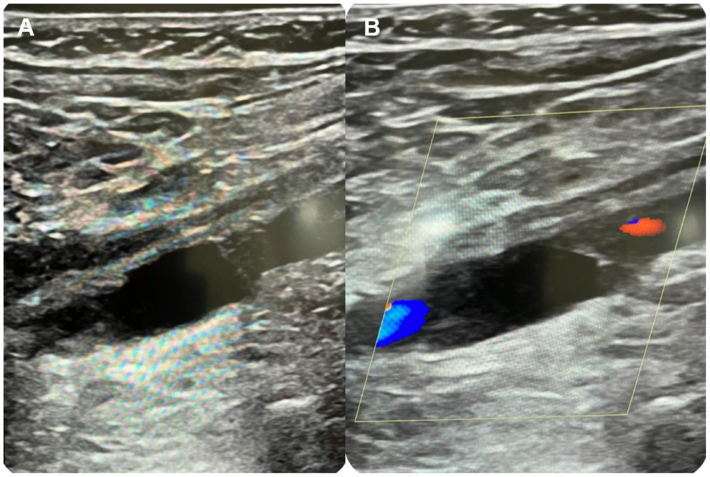
Duplex ultrasound (DUS) of the right popliteal artery confirming cystic adventitial disease (CAD). **(A)** Longitudinal B-mode image demonstrating an anechoic, cystic structure compressing the arterial lumen. **(B)** Color Doppler image showing no-flow within the compressed lumen due to extrinsic compression by the cyst.

**Fig 4:**
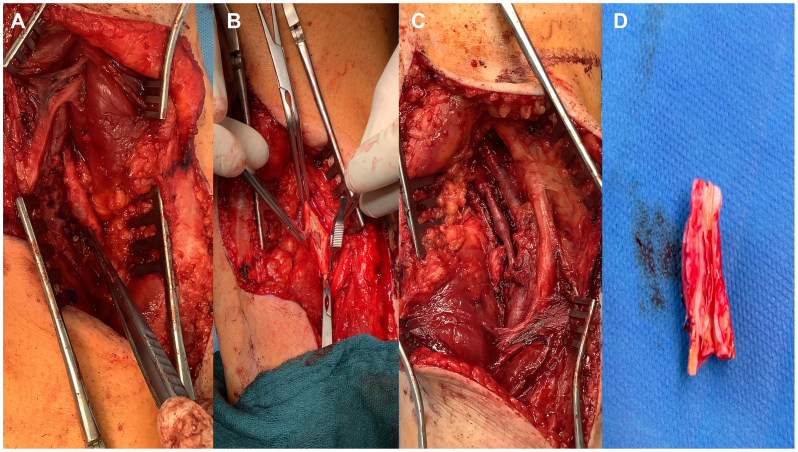
Intraoperative images of open surgical repair of cystic adventitial disease (CAD) of the popliteal artery. **(A)** Exposure of the popliteal artery through a posterior approach. **(B)** Resection of the diseased arterial segment. **(C)** Completion of femoropopliteal bypass with small saphenous vein interposition graft. **(D)** Resected popliteal artery segment demonstrating cystic degeneration.

Given these findings, the procedure was discontinued to avoid inappropriate endovascular treatment. Follow-up DUS confirmed the findings observed on IVUS (Fig 3). The patient subsequently underwent open surgical repair through a posterior approach, with resection of the affected popliteal segment and interposition femoropopliteal bypass using the small saphenous vein (Fig 4). Recovery was uneventful, with early mobilization and hospital discharge on postoperative day 5 on single antiplatelet therapy with aspirin 100 mg. At 1-month follow-up, the ABI had normalized to 1.0 on the operated side, DUS demonstrated a widely patent graft with triphasic distal waveforms, and the patient reported complete resolution of claudication symptoms. At 12-month follow-up, the patient remained asymptomatic with normal ABI values and DUS confirming continued graft patency.

Written informed consent was obtained from the patient for publication of this case report and accompanying images.

## Methods

We conducted a focused review of the literature to identify patients with CAD in whom the diagnosis was made via IVUS. Given the rarity of CAD diagnosed by IVUS, a focused literature review was performed rather than a full systematic or scoping review. A systematic search of PubMed and EMBASE was performed from January 1992 to July 2025, supplemented by manual screening of reference lists from relevant articles.

The following search phrases were used: “cystic adventitial disease AND popliteal AND intravascular ultrasound,” “adventitial cyst AND IVUS,” and related combinations (“intravascular ultrasonography” OR “endovascular ultrasound”). Titles and abstracts of all retrieved studies were screened. Full texts were reviewed when abstracts suggested potential eligibility.

**Fig 5:**
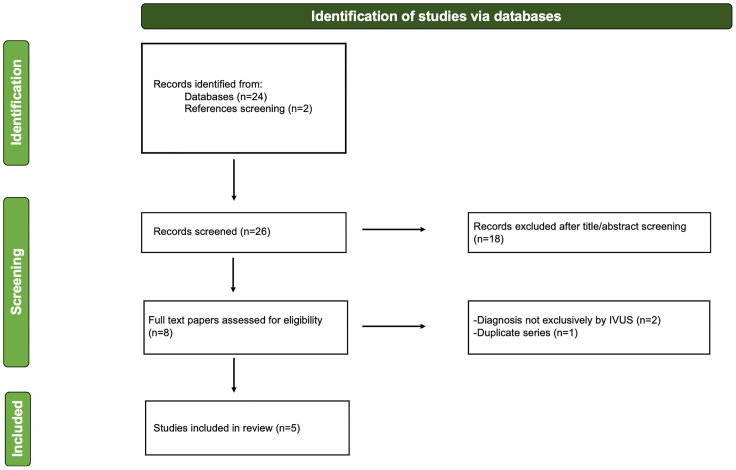
Preferred Reporting Items for Systematic reviews and Meta-Analyses (PRISMA)-style flow diagram illustrating the study selection process for the focused literature review. *IVUS*, Intravascular ultrasound.

We included case reports, case series, and observational studies that described popliteal CAD with IVUS imaging findings. Reviews without primary data, nonpopliteal cases, nonarterial cysts, and duplicate publications were excluded. Only articles with abstracts and/or full texts available in English were considered. The process of identification, screening, exclusion, and inclusion of studies is summarized in Fig 5.

## Results

The literature search yielded 26 articles, of which five case reports met the inclusion criteria and described the use of IVUS in the diagnosis of popliteal CAD.^10^, ^11^, ^12^, ^13^, ^14^ In total, five individual cases were identified between 1996 and 2025 (Table). Four patients were male, with a median age of 44 years (range, 34-87 years). The right popliteal artery was affected in three cases and the left in two cases. Two patients presented with at least one risk factor for atherosclerosis, whereas the others had no significant cardiovascular comorbidities. The clinical presentation was consistently IC. More detailed information was available for three patients, all of whom were categorized as Rutherford stage 3.

**Table:** Characteristics of the included studies

Author (year)	Age, years	Sex	Side	Atherosclerotic risk factors	Clinical presentation	Imaging prior to IVUS	Treatment	Outcome
Vos et al (1996)^10^	39	M	Right	Smoking	IC – Rutherford Stage 3	DSA	Aspiration/interposition saphenous vein graft	Symptom resolution
Koppensteiner et al (1996)^11^	34	F	Left	None	IC	DSA	Surgical	N/A
Foster et al (2001)^12^	44	M	Right	None	IC	DUS, DSA	Interposition saphenous vein graft	Symptom resolution
Niizeki et al (2016)^13^	87	M	Left	AH, DM, DLP, CKD	IC – Rutherford Stage 3	CO_2_ DSA	Interposition saphenous vein graft	Symptom resolution
Miyauchi et al (2023)^14^	64	M	Right	None	IC – Rutherford Stage 3	CTA, DSA	Conservative	Symptom resolution

*AH*, Arterial hypertension; *CKD*, chronic kidney disease; *CTA*, computed tomography angiography; *DLP*, dyslipidemia; *DM*, diabetes mellitus; *DSA*, digital subtraction angiography; *DUS*, duplex ultrasound; *IC*, intermittent claudication; *IVUS*, intravascular ultrasound; *N/A*, not available.

DSA was performed in all cases but did not establish the diagnosis. Computed tomography angiography in one patient and DUS in another also failed to detect CAD. Typical IVUS findings included hypoechoic, crescentic, or multiloculated cysts compressing the lumen with preservation of the intima–media complex.

Treatment approaches were heterogeneous. One patient (20%) was managed conservatively with spontaneous resolution and durable symptom-free follow-up at 7 years. Another patient (20%) was initially treated conservatively but subsequently required interposition grafting with autologous saphenous vein. One case (20%) underwent direct interposition vein grafting, whereas another (20%) was treated with repeated percutaneous aspirations followed by definitive interposition vein graft bypass due to symptom recurrence. In the remaining patient (20%), surgical treatment was performed, but no details regarding the procedure were available. Follow-up information was reported in four of the five cases, with durations ranging from the early postoperative period to 7 years. Patients who underwent definitive surgical excision with vein grafting remained symptom-free, and the single patient managed conservatively demonstrated sustained regression without recurrence at long-term follow-up. No recurrences were documented in any of the cases with available follow-up data. No major perioperative complications were reported in the reviewed cases. However, recurrence of symptoms occurred in one patient initially treated with percutaneous cyst aspiration, requiring subsequent surgical reconstruction.

## Discussion

CAD of the popliteal artery is a rare, nonatherosclerotic cause of claudication, affecting otherwise healthy middle-aged men.^1^, ^2^, ^3^ Accurate preoperative diagnosis is crucial, because outcomes depend on the excision of the cyst wall rather than simply restoring lumen patency.

IVUS is increasingly embedded in contemporary peripheral interventions, particularly for complex femoropopliteal disease.^15^ Beyond artery sizing and stent selection, IVUS refines subintimal vs intraluminal passage, characterizes calcification score, optimizes landing zones, and detects complications that intraoperative angiography usually misses.^9^^,^^16^^,^^17^ Although mandatory use is not yet guideline-codified and varies between centers, there is a clear trend toward broader adoption. Multidisciplinary expert consensus statements^18^ now advocate routine or near-routine IVUS use in complex infrainguinal lesions, and growing evidence suggests improved patency and fewer reinterventions, especially when treating TASC C/D lesions.^15^

By contrast, the diagnostic role of IVUS for nonatherosclerotic causes of IC is far less visible in the literature. Popliteal CAD is a classic masquerader of atherosclerotic PAD, whereas angiography shows a smooth, eccentric stenosis, which can mislead operators toward endovascular repair. The use of IVUS can unmask the true pathology, revealing an extraluminal, hypoechoic cyst compressing the lumen with preservation of intimal architecture. In retrospect, DUS may demonstrate features suggestive of CAD, including an anechoic or hypoechoic cystic structure adjacent to the arterial wall causing extrinsic compression of the lumen, typically in the absence of atherosclerotic plaque. In our case, however, the compressed lumen was initially interpreted as intraluminal thrombotic material. The rarity of CAD and its variable sonographic appearance may contribute to diagnostic misinterpretation, particularly when clinical suspicion is low.

Our focused literature review identified only a handful of cases of popliteal CAD in which IVUS contributes to diagnostic clarification when conventional imaging fails to provide clarity,^10^, ^11^, ^12^, ^13^, ^14^ underscoring how under-reported this application remains.

Similar to most published patients, our case involved a middle-aged man presenting with IC and angiographic findings suggestive of atherosclerotic occlusion. In all reports, IVUS was critical in revealing a cystic lesion with preservation of intima. Treatment strategies among the prior cases were heterogeneous, ranging from conservative management with spontaneous resolution^14^ to repeated aspirations followed by definitive vein grafting,^10^ and several reports of open resection with autologous vein interposition grafts.^11^, ^12^, ^13^ In one case, the operative details were not described.^11^ Follow-up, which extended up to 7 years, consistently demonstrated freedom from recurrence when surgical resection and reconstruction were performed. Our case aligns with this body of evidence by reinforcing the diagnostic utility of IVUS and the durability of open repair with vein grafting, while also emphasizing that reliance on angiography alone could have led to unnecessary and ineffective endovascular intervention.

IVUS should not replace DUS, computed tomography angiography, or magnetic resonance angiography, but may provide decisive clarification when an endovascular approach is already underway and lesion morphology is atypical. IVUS offers important advantages in atypical PAD presentations, particularly by helping differentiate atherosclerotic from nonatherosclerotic lesions. This distinction is critical, as durable outcomes depend on addressing the cyst wall rather than simply dilating the compressed lumen. Systematic reviews consistently report higher recurrence rates with cyst aspiration or plain balloon angioplasty alone, whereas surgical cyst excision, with or without vein graft reconstruction, yields better durability.^19^^,^^20^ In this context, IVUS can help prevent an endovascular-first approach by demonstrating when a stenosis or occlusion is due to extrinsic compression.

From a differential diagnosis standpoint, the combination of a young or low-risk patient with unilateral calf claudication, minimal atherosclerotic burden, and a smooth popliteal narrowing should trigger consideration of CAD or popliteal artery entrapment syndrome. In this context, IVUS offers decisive, catheter-based clarification when MRI or DUS is unavailable, equivocal, or discordant with angiography. A pragmatic approach is to consider IVUS in patients with characteristics not typical for PAD or a smooth, eccentric popliteal lesion inconsistent with the degree of atheromatosis. If IVUS confirms an adventitial cyst, the patient should be referred for surgical cyst excision, whereas endovascular tools are best reserved for highly selected situations. Finally, broader awareness and systematic reporting of IVUS findings in rare claudication etiologies are needed.

Our focused literature review was limited to PubMed and to English-language publications, which may have excluded relevant cases reported in other databases or languages. Additionally, the included literature consists of isolated case reports, with heterogeneous reporting of operative details and follow-up duration. Because the available evidence consists of isolated case reports, the findings of this focused literature review should be interpreted descriptively rather than quantitatively. Finally, given the rarity of IVUS-documented CAD, conclusions must be interpreted with caution. Larger multicenter registries or pooled collaborations would be required to better define recurrence rates and the role of IVUS in guiding therapy.

## Conclusions

This case report illustrates the potential adjunctive value of IVUS in clarifying the diagnosis of CAD in a patient with a chronic total popliteal artery occlusion initially presumed to be atherosclerotic. Recognition of this rare entity through advanced imaging prevented inappropriate endovascular intervention and facilitated timely surgical management, resulting in favorable clinical outcomes. By sharing this experience, we aim to raise awareness of CAD as a differential diagnosis in isolated popliteal artery occlusion and underscore the diagnostic value of IVUS in complex vascular cases. Overall, the focused literature review suggests that IVUS was consistently used as an adjunctive tool to aid diagnostic clarification of popliteal CAD in reported cases, particularly when conventional imaging was inconclusive or discordant.

## Funding

None.

## Disclosures

M.L. and K.S. have received speaker honoraria from Philips. The remaining author reports no conflicts.
